# Management of Neuromuscular Blocking Drug Extravasation With Sugammadex: A Case Report

**DOI:** 10.7759/cureus.115645

**Published:** 2026-09-02

**Authors:** William Crafton, Max Orlov, Warren Southerland

**Affiliations:** 1 Anesthesiology, Greater Baltimore Medical Center, Towson, USA; 2 Anesthesiology, Anesthesia Company LLC, Annapolis, USA

**Keywords:** intravenous extravasation, neuromuscular blockade drug reversal, neuromuscular blockade drugs, post-anesthesia care, sugammadex

## Abstract

Extravascular infiltration of neuromuscular blocking drugs (NMBDs) is a perioperative complication that may result in delayed and recurrent paralysis due to gradual systemic absorption from subcutaneous tissue.

We report the case of a 64-year-old female who experienced inadvertent peripheral intravenous infiltration (PIV) during intraoperative administration of vecuronium and rocuronium. Following routine reversal with sugammadex, she developed recurrent episodes of respiratory compromise and profound weakness in the post-anesthesia care unit, consistent with delayed absorption of the extravasated NMBD. Each episode resolved promptly after repeat intravenous administration of sugammadex.

This case highlights the unpredictable clinical course of NMBD extravasation and demonstrates that recurrent paralysis may occur despite initial successful reversal, requiring quantitative neuromuscular monitoring, extended postoperative observation, and administration of repeat doses of sugammadex as delayed systemic absorption occurs.

## Introduction

Inadvertent extravascular infiltration of neuromuscular blocking drugs (NMBDs) is an uncommon but potentially hazardous perioperative event. When an intended intravenous dose is deposited into surrounding tissue, a subcutaneous drug reservoir may form, resulting in a depot effect with delayed systemic absorption, unpredictable onset, and prolonged duration of neuromuscular blockade [1]. In contrast to the rapid and reliable pharmacokinetics expected after intravenous administration, these cases may present with failed or delayed intubating conditions initially, followed later by residual or recrudescent paralysis as the drug is gradually absorbed.

The available literature on this complication appears limited, including a review article detailing heterogeneous case reports, a case series, and a report associated with a clinical investigation conducted prior to the introduction of sugammadex [2]. Across published cases, rocuronium has been the most commonly implicated agent, with reports describing delayed onset of paralysis, prolonged postoperative monitoring, and frequent need for pharmacologic reversal or continued ventilatory support. Because absorption depends on factors such as injection site perfusion, patient habitus, or comorbid disease [3], the clinical course can be highly variable and difficult to predict.

Safe emergence from anesthesia after suspected NMBD infiltration therefore requires a management strategy that accounts for both residual blockade and the possibility of continued late absorption. Although quantitative neuromuscular monitoring is essential, the optimal postoperative management of patients following significant NMBD extravasation remains poorly defined, particularly regarding the duration of monitoring, risk of recurrent neuromuscular blockade after apparently successful reversal, and the role of repeat sugammadex administration. Sugammadex offers practical advantages for aminosteroid NMBDs, such as rocuronium and vecuronium, by rapidly encapsulating circulating NMBD and providing reliable reversal [4]. However, because sugammadex does not remove NMBD remaining within an extravascular tissue depot, its initial administration may not prevent recurrent blockade as additional drug is absorbed.

The present case illustrates this clinical knowledge gap by demonstrating three episodes of clinically significant recurrent respiratory muscle weakness following initial successful reversal with sugammadex after inadvertent vecuronium and rocuronium infiltration. Each episode resolved following repeat intravenous sugammadex administration, highlighting the potential for delayed, recurrent paralysis despite apparent initial recovery and the importance of prolonged postoperative monitoring. Given the absence of formal guidance regarding this scenario, this case provides a stepwise protocol of recognition, monitoring, and management of recurrent neuromuscular blockade following NMBD extravasation.

## Case presentation

A 64-year-old female with a history of hypertension, ulcerative colitis, and an asymptomatic heart murmur was admitted for surgical repair of an incarcerated ventral hernia by bilateral posterior sheath release with myofascial flap advancement. Prior to the procedure, anesthesia and surgical informed consents were obtained from the patient by the respective teams. A physician anesthesiologist was supervising a certified registered nurse anesthetist (CRNA) for the case. Intraoperatively, the patient received a continuous infusion of NMBD, vecuronium, into a peripheral intravenous (PIV) line in the left forearm. Train-of-four (TOF) counts were 0/4 after induction and 0/4 approximately one hour after induction.

Approximately two hours into the case, the patient began to breathe over the ventilator despite the NMBD infusion and had a TOF count of 1/4 at that time. The CRNA in the case subsequently administered rocuronium to achieve muscle paralysis, but the patient continued to have twitches and attempted to initiate respirations. After examining the PIV, the patient’s forearm was found to be edematous, erythematous, cool, and firm to the touch, consistent with findings of PIV extravasation. It was unknown when exactly this event occurred, but it was recorded that vecuronium (1600 mcg), rocuronium (60 mg), phenylephrine (300 mcg), and lactated Ringer’s (1200 mL) were administered via the PIV prior to its discontinuation. After palpating and confirming the presence of a left radial pulse, a new PIV was placed and used, and the procedure continued without complication.

The patient was found to have a TOF count of 0/4 just prior to emergence. The 4 mg/kg dose of sugammadex for deep blockade was chosen for reversal due to the uncertainty of the amount of NMBD administered intravenously due to the extravasation. The patient’s weight was 90.3 kg, and the weight-based dose of sugammadex for this patient was calculated to be 361.2 mg. The CRNA administered 400 mg of sugammadex for NMBD reversal, resulting in a TOF count of 4/4 with sustained tetany and subsequent extubation. The total duration of the procedure was 214 minutes.

Shortly after the patient arrived in the post-anesthesia care unit (PACU), the patient became hypopneic, lethargic, and sluggish, requiring bag-valve-mask resuscitation and complaining of surgical site pain. The last dose of opioid was hydromorphone 0.5 mg IV prior to emergence (total opioid consumption during the case was fentanyl 100 mcg IV and hydromorphone 1.5 mg IV). TOF was not performed due to the patient’s awareness. After evaluation by a physician anesthesiologist, another dose of sugammadex (200 mg, using the 2 mg/kg dose of sugammadex for moderate blockade) was administered via IV to resolve the symptoms from the extravasated NMBD.

This presentation of paralysis occurred two additional times, with approximately two hours between episodes during the patient’s stay in PACU, notably presenting as shortness of breath and inability to speak or raise her upper extremities, requiring repeat administration of 200 mg of sugammadex on each occasion. The infiltrated PIV site on the left forearm was found to be 15 cm in length, 13 cm in width, and 33 cm in circumference when examined in PACU. The anesthesia and surgical team agreed to admit the patient to the ICU for overnight monitoring for potential episodes of recurrent paralysis requiring reversal and/or airway management.

The patient did not require additional reversal during the overnight ICU stay, maintaining oxygen saturation >92% with oxygen via nasal cannula. The patient’s left forearm swelling due to the IV extravasation improved, and she did not have any additional paralytic events. The patient was downgraded from ICU status on POD#2 and discharged by the primary team on POD#3.

## Discussion

Extravascular infiltration of NMBDs presents a unique pharmacokinetic challenge. Rather than entering the circulation immediately, drug deposited within subcutaneous tissue forms a reservoir from which absorption occurs gradually over time [1]. This depot effect may initially manifest as failure to achieve adequate paralysis despite escalating NMBD doses, followed by delayed or recurrent neuromuscular blockade after apparent recovery. Caution should also be taken with patients who are elderly or have decreased renal function, as NMBD extravasation can mimic recurrent neuromuscular blockade due to poor clearance [5,6]. The timing and magnitude of absorption are unpredictable and are likely influenced by tissue perfusion, local blood flow, injection site, patient body habitus, and regional edema, making the duration of clinical risk difficult to estimate [3]. Furthermore, commonly identified risk factors for PIV infiltration include female gender, multiple attempts at cannulation, patient movement during cannulation, and placement into the hand, wrist, or antecubital fossa [7].

Our patient's presentation closely illustrates this depot effect. Persistent spontaneous respiratory effort despite administration of both a continuous vecuronium infusion and rocuronium bolus ultimately led to recognition of PIV infiltration. More notably, despite routine reversal with sugammadex and initial clinical recovery, she developed three distinct episodes of recurrent respiratory muscle weakness in the PACU over several hours. Each episode resolved rapidly following repeat intravenous sugammadex administration, suggesting continued systemic absorption of previously extravasated aminosteroid NMBD rather than incomplete initial reversal.

Sugammadex offers several theoretical and practical advantages in this setting. Unlike acetylcholinesterase inhibitors, sugammadex directly encapsulates circulating intravascular rocuronium and vecuronium molecules, producing rapid and predictable reversal independent of the depth of blockade [8]. However, in the case of an extravascular drug depot, newly absorbed NMBD may continue entering the circulation after the initial reversal dose has been administered. Consequently, recurrent paralysis may occur if additional circulating NMBD exceeds the amount of free sugammadex available for encapsulation. It has been reported that in the setting of a large subcutaneous depot, dosing sugammadex at 4 mg/kg to facilitate an excess of circulating sugammadex can help neutralize ongoing neuromuscular blockade [9]. Our case demonstrates that repeat administration of sugammadex can successfully treat recurrent neuromuscular blockade until the extravasated reservoir has been sufficiently absorbed and neutralized.

This experience also highlights several practical considerations for perioperative management. Failure to achieve expected paralysis after appropriate NMBD dosing should prompt immediate evaluation of intravenous access before administering additional doses. Once infiltration is identified, clinicians should anticipate delayed systemic absorption and avoid assuming that a successful initial reversal eliminates future risk. Quantitative neuromuscular monitoring, frequent respiratory assessment, and prolonged postoperative observation are critical. Patients with substantial suspected NMBD extravasation - particularly those experiencing recurrent weakness or respiratory compromise - may benefit from admission to a monitored setting such as an ICU or step-down unit where airway support and repeat sugammadex administration are readily available. Thus, based on this case and the available literature, we suggest the following practical approach, as seen in Table 1, when significant NMBD extravasation is suspected.

**Table 1:** Management Steps for Suspected Neuromuscular Blockade Drug Extravasation TOF: train-of-four; PACU: post-anesthesia care unit

	Management Steps for Suspected Neuromuscular Blockade Drug Extravasation
1	Recognize unexpected failure of paralysis and ensure stable vital signs. Assess for signs of inadequate neuromuscular blockade (breathing over ventilator, twitches on TOF).
2	Evaluate IV access. Inspect the IV site for swelling, blanching, or coolness and consider extravasation if suspicion arises.
3	Replace infiltrated IV access immediately. Discontinue use of the suspected IV and establish a new, reliable IV access at a different site.
4	Use quantitative neuromuscular monitoring throughout emergence. Confirm return of neuromuscular function and guide management decisions with objective monitoring (e.g., TOF).
5	Reverse with sugammadex rather than neostigmine when able. Administer sugammadex for prompt and reliable reversal of aminosteroid neuromuscular blockade.
6	Observe patients for recurrent weakness during postoperative period. Monitor for signs of residual/recurrent neuromuscular blockade in PACU and beyond.
7	Be prepared to administer repeat sugammadex if delayed paralysis recurs. Have a low threshold to re-dose sugammadex based on clinical signs and neuromuscular monitoring.
8	Consider overnight monitored admission after large-volume extravasation or recurrent paralysis. Ensure continued observation and respiratory monitoring in high-risk cases.

Although this report describes a single patient and definitive management recommendations cannot be established, it adds to the growing body of literature supporting extended monitoring following significant aminosteroid NMBD extravasation as well as success with repeated sugammadex administration. Additional multicenter case collection or prospective reporting would help define optimal observation periods, repeat sugammadex dosing strategies, and criteria for safe discharge.

## Conclusions

Extravascular infiltration of aminosteroid NMBDs is a rare but clinically significant complication that may produce delayed and recurrent paralysis due to unpredictable systemic absorption from a subcutaneous drug depot. This case demonstrates that successful initial reversal with sugammadex does not preclude subsequent episodes of neuromuscular blockade in the case of drug infiltration, and that repeat administration may be required as additional drug enters the circulation. Clinicians should maintain a high index of suspicion when neuromuscular blockade is unexpectedly delayed, promptly evaluate intravenous access, utilize quantitative neuromuscular monitoring, and provide extended postoperative observation in an appropriately monitored setting. Until formal guidelines become available, overnight monitoring with readiness for repeat sugammadex administration appears to be a reasonable and safe management strategy for patients with substantial suspected NMBD extravasation.
